# M. Lallemand on Visceral Abscesses (Purulent Depôts) after Traumatic Lesions

**Published:** 1829-01-01

**Authors:** 


### MONTPELLIER HOSPITAL.

#### I. VISCERAL ABSCESS (PURULENT DEPÔTS) AFTER TRAUMATIC LESIONS.

This curious and dangerous consequence of injuries and operations has hitherto been considered as rather rare. But now we can scarcely open a journal, or go into a surgical ward, between Ben Nevis and the Appennines, without meeting examples. We believe, it is now nearly as often seen at St. George's Hospital as erysipelas!

In the Ephemerides of Montpellier, (1828) we find a series of cases selected form the wards of M. Lallemand, where hepatic abscesses, as they are called, were found to succeed traumatic lesions of different kinds. It is curious, that the LIVER continues now, as in the time of Dessault, to be the organ most commonly affected in this way, after wounds or operations, on the Continent; whereas, the lungs are much more frequently the seat of the disease in this country. Can climate and constitution be the cause of this difference? We think there can be no other way of accounting for it. There is no just reason for accusing the pathologists, on either side of the channel, of want of accuracy in their observations. The respiratory apparatus, in our climate, is, unfortunately, but too much disposed to diseases of various kinds, and we imagine that, in this predisposition, will be found the solution of the discrepancy in question.

Hitherto, the diagnostic marks by which the formations of these dangerous depôts might be suspected, were considered as extremely obscure, and difficult of recognition. At present, M. Lallemand is said to be positive in his diagnosis of the malady; and, we believe, that the surgeons of our great hospitals here, are beginning to have little difficulty in foretelling the existence of pulmonary and hepatic abscesses, during the progress of the malady. We shall now glance at some of the cases recorded in the Ephemerides above-mentioned.

*Case* 1. A young soldier, 24 years of age, having been subject, for many years, to attacks of epilepsy, got drunk and fell on his head, the wound in the integuments of which was united by suture. Having complained of weight in his head, he was bled. The abdomen next became the seat of pain, and the usual antiphlogistic measures were employed. He now got better, and, while walking about in the court of the hospital, he caught cold, and became affected with erysipelas of the face—pain and tension of the right hypochondrium—general icteritious tint. It was found, on examination by the stethoscope, that there was inflammation on both sides of the chest—while other symptoms indicated hepatic inflammation. These phlogoses resisted the usual means —the head became affected—and the patient died. On dissection, the liver was found to contain depôts of purulent matter, lined with a polished membrane, the intervening hepatic parenchyma being softened. The appearances in the chest were such as had been suspected during life.

*Case* 2. A young soldier underwent a painful operation for a urethral fistula, after which his surface became icteritious, and he complained of tension in the right hypochondrium, with vomiting. These symptoms receded, when the sound was removed from the urethra—and they returned when the instrument was re-introduced. They were accompanied, this time, with vomitings of green and yellow bile, great alteration in the features—extreme tenderness in the region of the liver—quick and compressed pulse—cold chills —violent pain in the right knee. The patient died. On dissection, the liver was found in the same state as that described in the preceding case, the other viscera being sound. In the knee there was a collection of purulent matter.

*Case* 3. In this case, the patient had undergone several painful operations for the demolition of a stone in the bladder, on the plan of Amusat and others. But the symptoms, so unequivocal in the two former cases, were far from being very apparent here. There was neither tumefaction nor pain in the right hypochondrium, nor vomiting—but merely a slight icteritious tint on the surface, acute pain in the loins, and also in the right knee. Purulent depôts were found in the liver, exactly similar to those detected in the other two cases.

M. Lallemand considers a softened condition of the hepatic parenchyma, as a sure proof of the existence of previous inflammation—and indeed as the necessary consequence of the hepatic phlogosis. In the next stage of the said inflammation, there is a kind of purulent infiltration into the substance of the organ; so that, on squeezing a portion of its substance, matter will be seen to ooze out *guttatim*. In the third stage, the puriform secretion is found formed into distinct depôts, such as have been already described.—Journal de Progrés.

---

